# Urban surveillance of *Babesia* spp. in dogs and cats: molecular evidence from Changsha, China

**DOI:** 10.3389/fvets.2026.1862561

**Published:** 2026-06-25

**Authors:** Jingwei Quan, Ziran Mo, Xingyu Ruan, Xu Jiang, Junyan Li, Xiaoping Luo, Jia Wang, Wenbin Yang, Wei Hu

**Affiliations:** 1 College of Life Sciences, Inner Mongolia University, Hohhot, China; 2 Inner Mongolia Academy of Agricultural & Animal Husbandry Sciences, Hohhot, China; 3 Affiliated Hospital of Inner Mongolia Minzu University, Tongliao, China; 4 Institutes of Biomedical Sciences, Inner Mongolia University, Hohhot, China; 5 National Institute of Parasitic Diseases, Chinese Center for Disease Control and Prevention, Key Laboratory of Parasite and Vector Biology of China Ministry of Health, WHO Collaborating Centre for Tropical Diseases, Joint Research Laboratory of Genetics and Ecology on Parasite-Host Interaction, Chinese Center for Disease Control and Prevention, Fudan University, Shanghai, China; 6 Department of Infectious Diseases, Huashan Hospital, State Key Laboratory of Genetic Engineering, Ministry of Education Key Laboratory for Biodiversity Science and Ecological Engineering, Ministry of Education Key Laboratory of Contemporary Anthropology, School of Life Sciences, Fudan University, Shanghai, China

**Keywords:** *Babesia gibsoni*, *Babesia microti*, companion animal, haplotype, zoonoses

## Abstract

**Background:**

Babesiosis is a globally relevant tick-borne disease affecting both animals and humans. However, data on *Babesia* spp. in companion animals remain limited in Changsha, a rapidly urbanizing city in central China with high human–animal contact. This study addresses this gap by characterizing the prevalence and genetic diversity of *Babesia* spp. in local dogs and cats, providing insights into urban transmission dynamics and the public health relevance of *B. microti* detection in companion animals.

**Methods:**

A total of 1,258 blood samples from dogs and cats were collected in Changsha between 2021 and 2022. *Babesia* spp. were detected by nested PCR targeting the *SSU rRNA* gene, and phylogenetic and haplotype analyses were performed to assess genetic diversity and sequence relatedness among the identified isolates.

**Results:**

*Babesia* spp. were detected in 4.85% (61/1,258) of the samples, with infection rates of 5.19% in cats and 4.24% in dogs. *B. gibsoni* was the predominant species in both hosts, and phylogenetic analysis showed high sequence similarity to Asian canine strains. A total of 19 *B. gibsoni* haplotypes were identified, with Hap_1 being the most prevalent and distributed across multiple provinces. In addition, *B. microti* was detected in feline blood samples for the first time in China. These sequences clustered with tick-derived strains from the United States, and six haplotypes were identified, including Hap_4, a lineage previously reported in humans and ticks.

**Conclusion:**

This study presents the first molecular evidence of *B. microti* infection in pet cats in China, alongside the detection of *B. gibsoni* in both dogs and cats with notable haplotype diversity. The seasonal variation in infection rates, particularly the increase in autumn, suggests a potential influence of seasonal tick activity on transmission dynamics. These findings underscore the epidemiological relevance of companion animals in urban tick-borne piroplasm surveillance and support further investigation of local tick vectors and reservoir hosts, particularly in relation to the potential public health relevance of *B. microti*.

## 1 Introduction

Babesiosis is a tick-borne hemoprotozoan disease that poses a serious threat to both human and animal health. It is primarily caused by apicomplexan parasites of the genus *Babesia*, which invade host erythrocytes and cause symptoms such as anemia, fever, jaundice, hemoglobinuria, and even death in severe cases (1–4). A global meta-analysis estimated the prevalence of human babesiosis at 2.23%, with rates reaching 4.17% in parts of Europe (5), and recent surveillance data indicate a rising incidence in the United States (6). Although global mortality data remain limited, the disease poses significant risks to immunocompromised individuals, the elderly, and splenectomized patients (7). With increasing global travel and cross-border animal movement, the risk of transregional transmission is growing, underscoring the urgent need for enhanced surveillance and early detection strategies.

Since their first identification by Babes in 1888 (8), over 100 *Babesia* species have been recognized worldwide, among which only a limited number have been documented as human-infective species, including *B. microti, B. venatorum, B. duncani*, and *B. divergens*, and have been implicated in human infection (6, 9, 10). *B. microti* is predominantly endemic in the northeastern and midwestern United States, where it accounts for the majority of human babesiosis cases. In China, sporadic human infections have also been reported in provinces such as Yunnan, Zhejiang, Taiwan and Xinjiang (11). Alongside this, companion animals such as dogs and cats are increasingly regarded as susceptible hosts and potential sentinels for local exposure to tick-borne piroplasms, although their reservoir competence for specific *Babesia species* requires further investigation.

In China, molecular evidence of *B. canis, B. gibsoni*, and *B. vogeli* has been reported in dogs from multiple provinces, including Henan, Shaanxi, Jiangsu, Hubei, and Hunan (12–16). However, compared to livestock and wildlife, systematic molecular studies on *Babesia* spp. in companion animals, particularly cats, remain limited. The prevalence, genetic diversity, and phylogeographic patterns of *Babesia* spp. in urban pet populations are still poorly characterized. Notably, although *B. microti* is a recognized zoonotic species, no molecular detection in domestic cats has been reported in China to date. This knowledge gap limits our understanding of the occurrence, genetic diversity, and potential ecological circulation of zoonotic *Babesia* species in urban companion-animal populations.

Changsha, a rapidly urbanizing city in central China, represents an epidemiologically relevant setting for tick-borne pathogen surveillance due to its dense pet population, favorable subtropical climate for tick activity, and increased overlap between human, animal, and peri-urban ecological interfaces. These ecological and demographic factors may jointly contribute to the establishment and spread of *Babesia* spp. infections (17–19). Therefore, Changsha provides a suitable urban setting for investigating the molecular epidemiology of tick-borne piroplasms in companion animals. Nevertheless, the epidemiological status and genetic characteristics of *Babesia* spp. in dogs and cats in Changsha remain unknown, limiting our understanding of their local transmission dynamics.

To address this gap, this molecular epidemiological study investigated *Babesia* spp. infections in dogs and cats in Changsha. Broad-range Piroplasmida PCR combined with *Babesia*-specific amplification and sequence analysis was used to improve molecular detection reliability and clarify the species composition and epidemiological characteristics of *Babesia* spp. in companion animals. Phylogenetic and haplotype network analyses further characterized the genetic diversity and sequence relatedness of circulating *Babesia* species, providing a basis for regional risk assessment and targeted control of tick-borne protozoan infections.

## 2 Materials and methods

### 2.1 Sample collection

In September 2021 and March 2022, a total of 1,258 blood samples were obtained from pet animals presented to animal hospitals in Changsha, Hunan Province, China, including 448 dogs and 810 cats. Blood samples were collected in EDTA-coated vacutainer tubes and transported to the laboratory in iceboxes, then stored at 4 °C until DNA extraction. As this study was based on available hospital-derived blood samples rather than a prospective questionnaire-based survey, detailed individual-level information, including age, sex, breed, exact residential location, clinical signs, tick infestation history, indoor/outdoor lifestyle, and hunting behavior, was not systematically recorded in the hospital records at the time of sampling. Therefore, these variables were not included in this epidemiological risk-factor analysis.

### 2.2 DNA extraction and PCR amplification

#### 2.2.1 DNA extraction

Genomic DNA was extracted from 100 μl of each blood sample using the DNeasy^®^ Blood & Tissue Kit (Qiagen, Hilden, Germany), according to the manufacturer's instructions. The concentration and purity of the extracted DNA were assessed using a NanoDrop spectrophotometer by measuring the A260/A280 ratio. Extracted DNA was stored at −20 °C until further molecular analysis.

#### 2.2.2 PCR amplification

Nested PCR targeting the *SSU rRNA* gene was performed for the broad-range detection of Piroplasmida (16, 20–24). The first round was performed using primers Piro1-S and Piro3-AS, and the second round was performed using primers Piro-A and Piro-B. Piroplasmida-positive samples were further examined using *Babesia*-specific nested PCR, with primers Bab5 and Bab8 in the first round and Bab6 and Bab7 in the second round. Primer sequences and expected product sizes are listed in Table 1.

**Table 1:** The oligonucleotide primers for identification of *Babesia* spp.

Gene	Identification code	Sequences (5^′^-3^′^)	Length (bp)	Specificity
*SSU rRNA*	Piro1-S	CTT GAC GGT AGG GTA TTG GC	408	*Piroplasmida*
	Piro3-AS	CCT TCC TTT AAG TGA TAA GGT TCAC		
	Piro -A	AAT ACC CAA TCC TGA CAC AGG G		
	Piro -B	TTA AAT ACG AAT GCC CCC AAC		
*SSU rRNA*	Bab 5	AAT TAC CCA ATC CTG ACA CAG G	400	*Babesia spp*.
	Bab 8	TTT CGC AGT AGT TCG TCT TTA ACA		
	Bab 6	GAC ACA GGG AGG TAG TGA CAA GA		
	Bab 7	CCC AAC TGC TCC TAT TAA CCA TTA C		

Each PCR reaction was performed in a final volume of 25 μl containing 12.5 μl of 2 × PCR Master Mix, 1.0 μl of each primer, 2.0 μl of DNA template, and nuclease-free water to the final volume. For the second-round PCR, 1.0 μl of the first-round PCR product was used as the template. Positive controls consisted of *B. microti* genomic DNA, and distilled water was used as the negative control in each PCR run. PCR-positive samples were retested in triplicate to confirm the reproducibility of the amplification results.

#### 2.2.3 PCR conditions

For broad-range detection of Piroplasmida, PCR amplification was performed under the following conditions: initial denaturation at 94 °C for 3 min; followed by 35 cycles of denaturation at 94 °C for 60 s, annealing at 55 °C for 60 s, and extension at 72 °C for 60 s; with a final extension at 72 °C for 10 min. For *Babesia*-specific detection, PCR was performed at 95 °C for 5 min, followed by 35 cycles of 95 °C for 30 s, 55 °C for 30 s, and 72 °C for 60 s, with a final extension at 72 °C for 7 min.

#### 2.2.4 Amplicon detection and visualization

PCR products were separated by electrophoresis on 1.0% agarose gels stained with nucleic acid dye. Amplicon sizes were verified using a 2,000 bp DNA ladder marker. Bands of the expected size were visualized under ultraviolet illumination and considered PCR-positive.

#### 2.2.5 Sequencing and sequence analysis

All PCR-positive amplicons generated by broad-range Piroplasmida PCR and *Babesia*-specific PCR were purified and subjected to bidirectional Sanger sequencing by BGI Genomics Co., Ltd. Forward and reverse sequences were assembled and manually checked to obtain high-quality consensus sequences. The obtained sequences were compared with reference sequences available in GenBank using the BLAST tool on the NCBI website (blast.ncbi.nlm.nih.gov/Blast.cgi) to confirm species identity. All validated nucleotide sequences were submitted to the GenBank database under accession numbers OQ804561 – OQ804621.

### 2.3 Haplotype network analysis and phylogenetic reconstruction

In this study, the *SSU rRNA* gene sequences of *Babesia* spp. (*B. microti* and *B. gibsoni*) were analyzed to investigate their genetic structure. Phylogenetic trees were constructed using MEGA11 (www.megasoftware.net) with the Maximum Likelihood method (ML). For tree construction, *B. conradae* (MH143374.1) and *B. microti* (MF401441.1) were used as outgroups. The Kimura two-parameter model was used as the nucleotide substitution model, with a gamma distribution to account for among-site rate variation. The robustness of the inferred topologies was assessed using 1,000 bootstrap replicates (25).

Additionally, *SSU rRNA* gene sequences of *B. microti* and *B. gibsoni* obtained in this study were combined with reference sequences retrieved from GenBank to form species-specific datasets. Genetic diversity was analyzed using DnaSP 6, which categorized the aligned sequences and generated haplotype data. Haplotype network reconstruction was performed using PopART (version 1.7, Dunedin, New Zealand) based on a statistical parsimony algorithm with a connection probability threshold of 95%, and the results were visualized as TCS haplotype networks (26).

### 2.4 Statistical analysis

Differences in the positivity rates of *Babesia* spp. infections among different host species and across seasons were assessed using the chi-square test. Fisher's exact test was used when expected cell counts were less than 5. A *P*-value < 0.05 was considered statistically significant. All statistical analyses were performed using SPSS version 21.0 (IBM Corp., Armonk, NY, USA) (27).

## 3 Results

### 3.1 Nucleotide sequence analysis

Nested PCR targeting Piroplasmida identified 61 positive samples, all of which were further confirmed as *Babesia* spp. by *Babesia*-specific amplification and sequencing. Sequence alignment of 61 *Babesia SSU rRNA* gene sequences obtained in this study revealed that 53 sequences (34 from cats and 19 from dogs) (GenBank accession numbers: OQ804569 - OQ804621) shared up to 99.72% sequence homology with the reference strain *B. gibsoni* (MH620203.1) and were identified as *B. gibsoni* (Supplementary Figure 1A). Eight sequences (GenBank accession numbers: OQ804561 - OQ804568) were obtained from cat samples, which exhibited up to 99.98% sequence identity with the reference sequence *B. microti* (MG199174.1), supporting their classification as *B. microti* (Supplementary Figure 1B). No other *Babesia* species were identified in this study.

### 3.2 Prevalence, species distribution and seasonal data of *Babesia* spp.

Blood samples from dogs and cats were collected at two distinct time points, September 2021 and March 2022, representing autumn and spring sampling periods, respectively. These two periods were selected to capture seasonal differences in tick activity that may influence the transmission of tick-borne piroplasms. In the spring of 2022, a total of 841 samples were collected, including 538 from cats and 303 from dogs. In the autumn of 2021, 417 samples were collected, comprising 272 from cats and 145 from dogs.

The overall prevalence of *Babesia* spp. was 4.85% (61/1,258), with 5.19% (42/810) in cats and 4.24% (19/448) in dogs. No significant difference was observed between cats and dogs (χ^2^ = 0.557, *P* > 0.05). Species distribution analysis showed that *B. gibsoni* was the predominant species, accounting for 53 positive samples, whereas *B. microti* was detected in eight cat samples. Among cats, 4.20% (34/810) were positive for *B. gibsoni* and 0.99% (8/810) for *B. microti*, whereas only *B. gibsoni* was detected in dogs (Table 2).

**Table 2:** Comparison of *Babesia* spp. infections in cats and dogs by PCR methods.

Season	Host	No. specimens	No. positive for *B. microti* (%)	No. positive for *B. gibsoni* (%)	No. positive for *Babesia* spp. (%)	OR (95% CI)	*P*-value
March, 2022	Cat	538	1 (0.19)	2 (0.37)	3 (0.56)	0.3342 (0.08805–1.263)	0.1448
	Dog	303	0 (0)	5 (1.65)	5 (1.65)		
	Total	841	1 (0.12)	7 (0.83)	8 (0.95)		
September, 2021	Cat	272	7 (2.57)	32 (11.76)	39 (14.34)	1.566 (0.8263–2.935)	0.2167
	Dog	145	0 (0)	14 (9.65)	14 (9.65)		
	Total	417	7 (1.68)	46 (11.03)	53 (12.71)		
March vs. September	Overall	1,258	8 (0.64)	53 (4.21)	61 (4.85)	0.06596 (0.03105–0.1401)	*P* < 0.05

Seasonal analysis showed that the overall prevalence of *Babesia* spp. was 0.95% (8/841) in March 2022, including 0.56% (3/538) in cats and 1.65% (5/303) in dogs. In contrast, the prevalence in September 2021 was 12.71% (53/417), including 14.34% (39/272) in cats and 9.65% (14/145) in dogs, which was significantly higher than that in March 2022 (χ^2^ = 83.541, *P* < 0.05). These results indicate that *Babesia* spp. infection rates did not differ significantly between host species, whereas a marked seasonal variation in prevalence was observed.

### 3.3 Phylogenetic relationship and haplotype analysis of *B. microti*

To elucidate the genetic structure of the *B. microti* isolates detected in local companion animals, phylogenetic analysis was performed on the *SSU rRNA* gene sequences obtained from positive samples. Haplotype analysis was further conducted to assess the diversity and sequence relatedness of *B. microti* strains.

**Figure 1:**
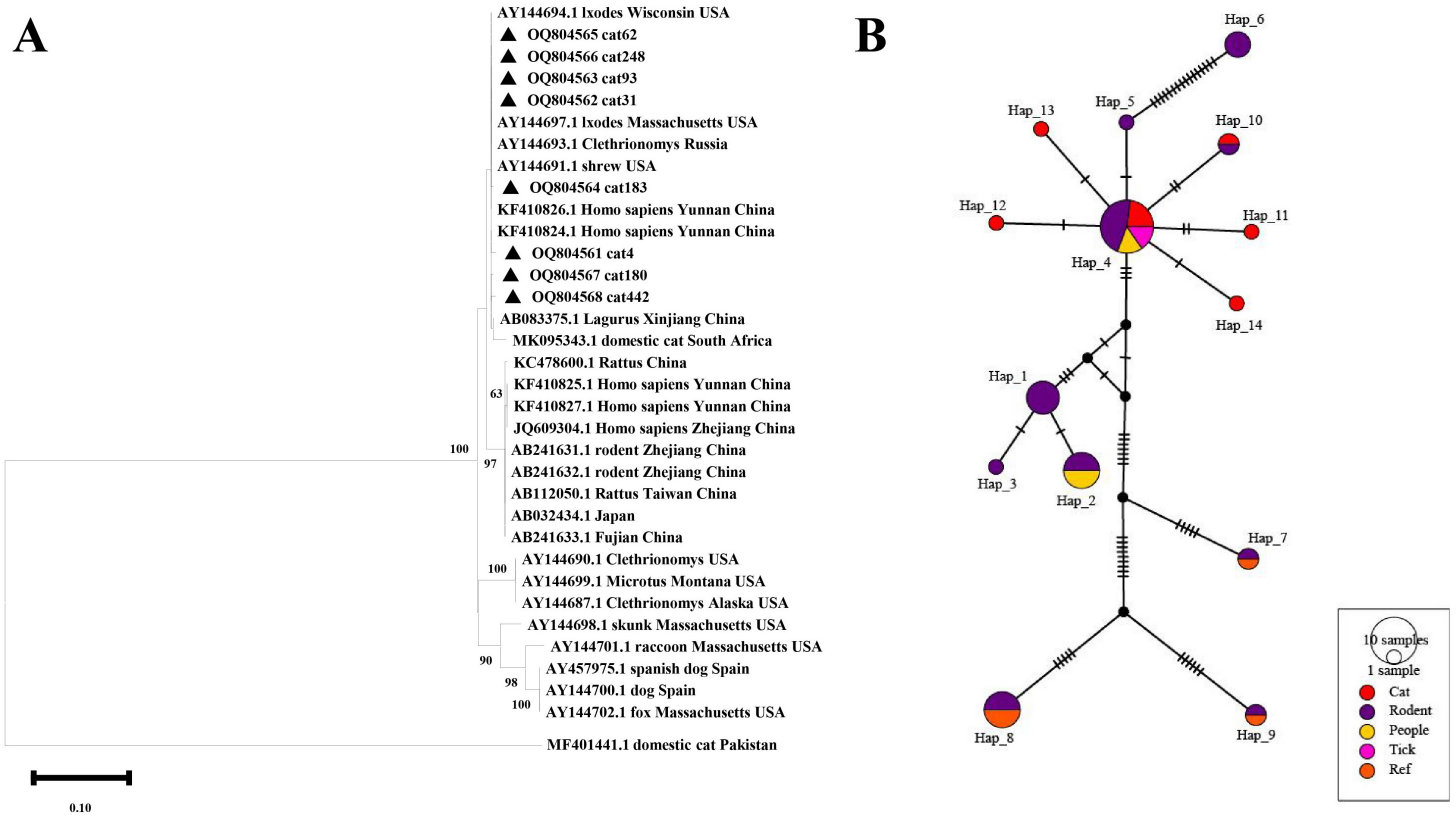
Phylogenetic tree and haplotype analysis of *B. microti*. **(A)** Phylogenetic tree of the *B. microti SSU rRNA* gene. Representative isolates from this study are marked with a triangle (▴). **(B)** Haplotype analysis results of *B. microti* sequences detected in this study and published *B. microti* sequences. Different colours represent different groups, including Cat, Rodent, People, Tick, and other regions, and the size of the circles represents the number of haplotypes.

For phylogenetic reconstruction, *SSU rRNA* sequences of *B. microti* from various geographic regions were retrieved from the GenBank database and analyzed alongside the eight sequences obtained in this study. These sequences were clustered with those derived from South African cats, Xinjiang rodents, Yunnan humans, and American ticks, indicating close genetic relatedness among *B. microti* sequences from different geographic regions and host sources. In contrast, they exhibited a greater genetic distance from *B. microti* strains reported in cats from Pakistan, suggesting regional sequence divergence (Figure 1A).

To further examine haplotype structure, a total of 14 haplotypes were identified based on *SSU rRNA* sequence alignments integrating data from GenBank and this study. Hap_4 accounted for 28.9% of the total sequences and was identified as the dominant haplotype. Six haplotypes (Hap_4, Hap_10, Hap_11, Hap_12, Hap_13, and Hap_14) were represented by sequences obtained in this study. Notably, Hap_4 was identified in both feline sequences from this study and previously reported human- and tick-derived sequences, suggesting the occurrence of closely related *B. microti* variants across different host sources (Figure 1B).

In summary, the *B. microti* strains identified in this study show conserved *SSU rRNA* sequence profiles and haplotype patterns, suggesting genetic relatedness to previously reported *B. microti* sequences from different host sources.

### 3.4 Phylogenetic relationship and haplotype analysis of *B. gibsoni*

**Figure 2:**
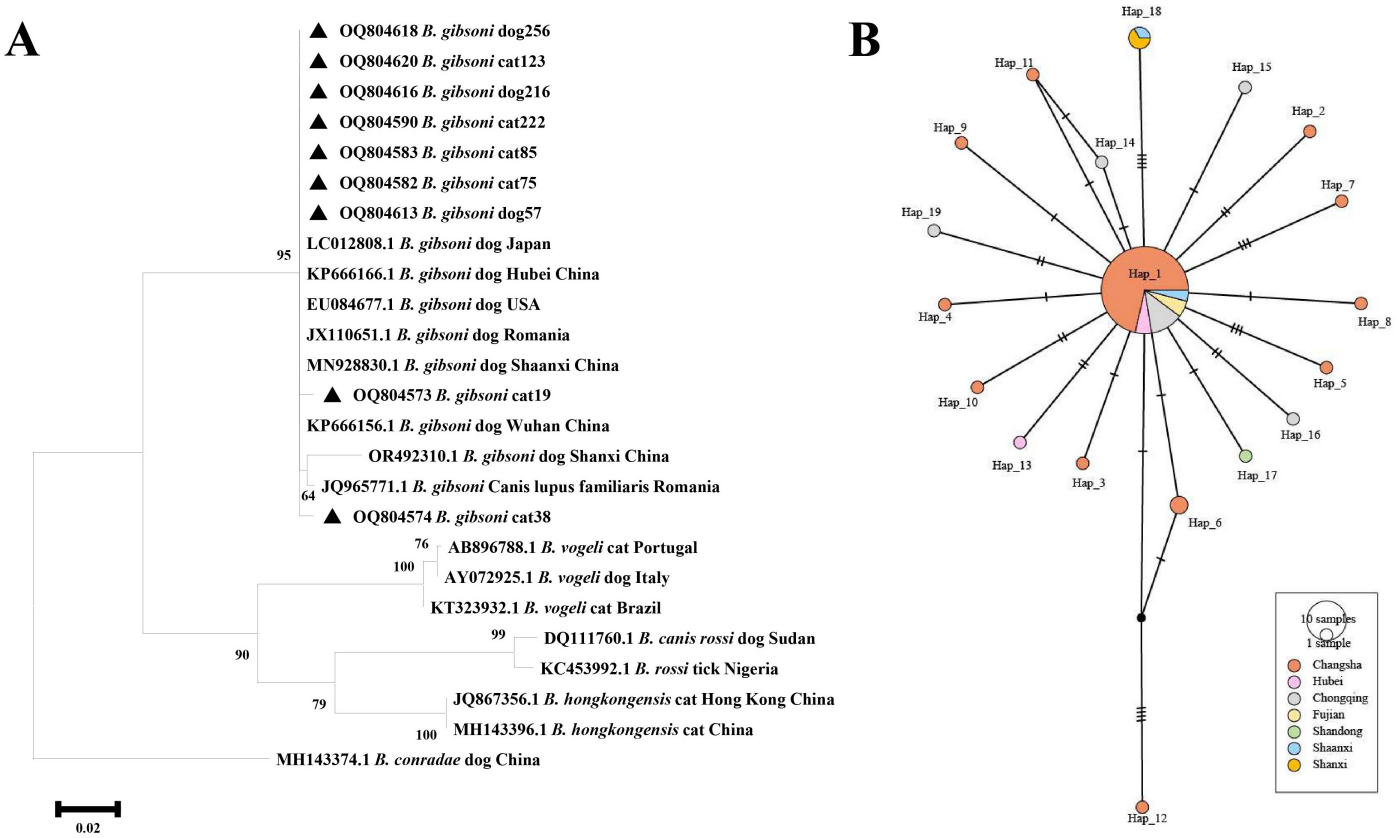
Phylogenetic tree and haplotype analysis of *B. gibsoni*. **(A)** Phylogenetic tree of the *B. gibsoni SSU rRNA* gene. Representative isolates from this study are marked with a triangle (▴). **(B)** Haplotype analysis results of *B. gibsoni* sequences detected in this study and published *B. gibsoni* sequences. Different colours represent different groups, including Changsha, Hubei, Chongqing, Fujian, and other regions, and the size of the circles represents the number of haplotypes.

Multiple sequence alignment was conducted on the 53 *B. gibsoni SSU rRNA* gene sequences obtained in this study. Based on nucleotide variation, nine representative sequences were selected for phylogenetic analysis. These sequences were clustered within the same clade as *B. gibsoni* reference strains from other regions, indicating close genetic relatedness at the *SSU rRNA* locus. Moreover, the strains identified from both canine and feline hosts showed a high degree of genetic similarity, suggesting conserved *SSU rRNA* sequence profiles among isolates from the two host species (Figure 2A).

To further characterize the genetic diversity and geographic distribution of *B. gibsoni, SSU rRNA* sequences from different provinces in China were retrieved from the GenBank database and incorporated into a haplotype network analysis. A total of 19 haplotypes were identified. Among these, two haplotypes were reported in Hubei (Hap_1 and Hap_13), two in Shaanxi (Hap_1 and Hap_18), five in Chongqing (Hap_1, Hap_14, Hap_15, Hap_16, and Hap_19), and one each in Fujian, Shandong, and Shanxi (Hap_1, Hap_17, and Hap_18, respectively). 12 haplotypes (Hap_1 to Hap_12) were derived from the current study. Notably, Hap_1 was centrally located within the network and accounted for approximately 70% of all analyzed sequences, indicating its predominance among the local *B. gibsoni* population. The remaining haplotypes reflected the presence of additional genetic variants among the Changsha isolates, demonstrating a certain level of genetic heterogeneity (Figure 2B).

Overall, *B. gibsoni* strains in dogs and cats from Changsha show high genetic conservation with co-circulating strains in Asia, yet the observed haplotypic diversity reflects the presence of multiple local sequence variants. These features suggest a relatively conserved *SSU rRNA* profile with detectable haplotypic variation among the *B. gibsoni* sequences analyzed in this study.

## 4 Discussion

*Babesia* spp. are tick-borne hemoprotozoan parasites that infect a wide range of vertebrate hosts, among which *Babesia microti, Babesia venatorum*, and related species have been confirmed to possess zoonotic potential (28, 29). Companion animals such as dogs and cats can serve as hosts for *Babesia* spp. and live in close contact with humans. The detection of the zoonotic species *B. microti* in these animals suggests the need to further investigate local tick vectors and potential reservoir hosts, raising possible public health concerns. As a rapidly urbanizing city with dense pet populations and complex peri-urban ecotones, Changsha provides a representative setting for investigating the ecology and transmission patterns of tick-borne protozoa under subtropical conditions. In this study, we applied molecular diagnostics, phylogenetic analysis, and haplotype network reconstruction to characterize *Babesia* spp. in dogs and cats. Our findings contribute baseline data for monitoring *Babesia* spp. in urban companion animals and support the value of continued surveillance in Changsha and similar subtropical urban settings.

This study presents the first molecular confirmation of *B. microti* in cats in China, providing new evidence for the occurrence of this zoonotic species in feline hosts. Although *B. microti* has been reported in rodents, ticks, and humans in regions such as Yunnan, Zhejiang, and Xinjiang (11, 30), its occurrence in feline hosts had not previously been documented in China. Notably, the haplotype (Hap_4) identified in cats was identical to that found in human- and tick-derived sequences, suggesting that closely related *B. microti* variants may circulate among different host sources. This finding highlights the need for integrated surveillance at the human-animal-vector interface to evaluate the potential transmission risk of *B. microti* in urban environments. Internationally, *B. microti* infection rates in cats have been reported at 13.2% in Pakistan (31), 2.4% in Turkey (32), and 0.8% in northern Italy (33), suggesting considerable variation across ecological contexts. In contrast, our study identified a lower infection rate (0.99%) in cats from Changsha, but its co-occurrence with human-related haplotypes underscores its potential public health relevance, especially in subtropical urban ecosystems. Given these findings, systematic molecular identification of ticks parasitizing pets in the region, along with human seroepidemiological screening, will help clarify the local transmission cycle of *B. microti*.

In addition to *B. microti, B. gibsoni* was identified as the dominant species in both cats and dogs. The infection rate in dogs (4.24%) was higher than those reported in Hainan (0.1%) (34) and Anhui (2.22%) (35), but lower than the prevalence observed in Hubei Province (32.00%) (15). In cats, the infection rate (4.20%) was comparable to Chongqing (4.3%), and higher than that reported in Fujian (3.1%) and Shandong (2.0%) (15), highlighting regional variation in the epidemiology of *B. gibsoni*. Although previous studies have reported *B. canis* and *B. vogeli* infections in dogs from Hunan Province (16), neither species was detected in the present canine blood samples. This discrepancy may be related to differences in sampling period, host exposure history, local tick communities, and molecular detection methods. Therefore, the absence of *B. canis* and *B. vogeli* in this survey does not exclude their circulation in Changsha, but may suggest low prevalence, focal transmission, or uneven distribution in the sampled dog population. Phylogenetic and haplotype analyses revealed that Hap_1 was the predominant haplotype, widely shared across multiple provinces, suggesting broad geographic distribution of this *B. gibsoni* haplotype in China. The detection of multiple haplotypes within the Changsha region further suggests a certain degree of local haplotypic diversity at the *SSU rRNA* locus. These findings underscore the importance of molecular epidemiological surveillance and targeted vector control measures, particularly in regions characterized by dense pet populations and tick-friendly climatic conditions.

Notably, no significant difference in *Babesia* spp. infection rates was observed between cats and dogs, suggesting that dogs and cats may have comparable exposure to infected ticks or similar environmental risk factors in the sampled population. The concurrent detection of *B. gibsoni* in both hosts may reflect shared exposure to tick vectors or overlapping living environments, although direct evidence for cross-species transmission was not obtained in this study. Seasonally, the significantly higher prevalence in autumn is consistent with increased seasonal tick activity. This seasonal trend is likely driven by increased vector abundance and higher frequency of tick-host interactions during favorable environmental conditions, reinforcing the need for timely vector monitoring and preventive interventions during periods of elevated transmission risk. However, these epidemiological interpretations should be considered in light of the limited individual-level information available in this study. Variables such as age, sex, breed, clinical status, tick exposure, indoor/outdoor lifestyle, and hunting behavior may influence the risk of *Babesia* infection in dogs and cats. Future longitudinal studies combining animal demographic data, clinical records, lifestyle questionnaires, tick identification, and wildlife surveillance are needed to better evaluate the risk factors and transmission pathways of *Babesia* spp. in urban companion animals.

In summary, this study provides baseline molecular evidence of *Babesia* spp. infection and genetic diversity in companion animals in Changsha, with the first detection of *B. microti* in cats in China suggesting its potential public health relevance. Despite being limited to a single urban region, the integration of molecular epidemiology, phylogenetic inference, and haplotype analysis strengthens the reliability of our findings. These results provide a foundation for future comparative studies across climatic zones and host populations, and inform the development of surveillance strategies tailored to urban pet ecosystems. Together, these findings support continued surveillance of tick-borne protozoa in Changsha and similar subtropical urban settings, and underscore the importance of routine screening in veterinary practice to support early detection, control, and public health preparedness.

## 5 Conclusion

This study confirmed the occurrence of *Babesia* spp. infections in dogs and cats in Changsha through molecular diagnostic analyses. Notably, *Babesia microti* was reported for the first time in pet cats in China. Although its prevalence was low, the detection of this recognized zoonotic species indicates its potential public health significance. The detection of both *B. gibsoni* and *B. microti* across species highlights their broad host range. Moreover, the significantly higher infection prevalence observed in autumn compared with spring suggests a seasonal pattern that may be associated with seasonal variation in tick activity and tick-host contact. These findings enhance our understanding of *Babesia* circulation in urban subtropical settings and underscore the importance of targeted surveillance in companion animals. However, the lack of detailed demographic, clinical, and lifestyle data limited host-level risk assessment, and future prospective studies incorporating standardized questionnaires and paired tick sampling are warranted.

## Data Availability

All sequences of the SSU rRNA gene obtained from the positive samples in the present study were deposited in the GenBank database under the accession numbers OQ804561 - OQ804621 (SSU rRNA gene).
